# Pulmonary vascular reactivity in growth restricted fetuses using computational modelling and machine learning analysis of fetal Doppler waveforms

**DOI:** 10.1038/s41598-024-54603-x

**Published:** 2024-03-11

**Authors:** Kilian Vellvé, Patricia Garcia-Canadilla, Mariana Nogueira, Lina Youssef, Angela Arranz, Ayako Nakaki, David Boada, Isabel Blanco, Rosa Faner, Francesc Figueras, Àlvar Agustí, Eduard Gratacós, Francesca Crovetto, Bart Bijnens, Fàtima Crispi

**Affiliations:** 1 BCNatal Fetal Medicine Research Center (Hospital Clínic and Hospital Sant Joan de Déu), University of Barcelona, Sabino Arana 1, 08028 Barcelona, Spain; 2 Institut d’Investigacions Biomèdiques August Pi i Sunyer (IDIBAPS), Barcelona, Spain; 3 Interdisciplinary Cardiovascular Research Group, Institut de Recerca Sant Joan de Déu, Esplugues de Llobregat, Barcelona, Spain; 4 Pneumology Department, Respiratory Institute, Hospital Clínic, University of Barcelona, Barcelona, Spain; 5 Centre for Biomedical Research on Respiratory Diseases (CIBER-ES), Madrid, Spain; 6 Cathedra Salud Respiratoria, University of Barcelona, Barcelona, Spain; 7 Centre for Biomedical Research on Rare Diseases (CIBER-ER), Madrid, Spain; 8 ICREA, Barcelona, Spain

**Keywords:** Respiratory tract diseases, Risk factors, Ultrasonography, Pregnancy outcome

## Abstract

The aim of this study was to investigate the pulmonary vasculature in baseline conditions and after maternal hyperoxygenation in growth restricted fetuses (FGR). A prospective cohort study of singleton pregnancies including 97 FGR and 111 normally grown fetuses was carried out. Ultrasound Doppler of the pulmonary vessels was obtained at 24–37 weeks of gestation and data were acquired before and after oxygen administration. After, Machine Learning (ML) and a computational model were used on the Doppler waveforms to classify individuals and estimate pulmonary vascular resistance (PVR). Our results showed lower mean velocity time integral (VTI) in the main pulmonary and intrapulmonary arteries in baseline conditions in FGR individuals. Delta changes of the main pulmonary artery VTI and intrapulmonary artery pulsatility index before and after hyperoxygenation were significantly greater in FGR when compared with controls. Also, ML identified two clusters: A (including 66% controls and 34% FGR) with similar Doppler traces over time and B (including 33% controls and 67% FGR) with changes after hyperoxygenation. The computational model estimated the ratio of PVR before and after maternal hyperoxygenation which was closer to 1 in cluster A (cluster A 0.98 ± 0.33 vs cluster B 0.78 ± 0.28, p = 0.0156). Doppler ultrasound allows the detection of significant changes in pulmonary vasculature in most FGR at baseline, and distinct responses to hyperoxygenation. Future studies are warranted to assess its potential applicability in the clinical management of FGR.

## Introduction

Fetal growth restriction (FGR) is a prevalent prenatal condition which has short- and long-term consequences in the neurodevelopment^1^ and cardiometabolic health of the individuals^2–4^. However, while the effect of FGR on the cardiovascular and brain development has been widely studied, there is less evidence regarding its effect on other organs such as the lungs. Some evidence of a deleterious effect of in utero growth restriction on lung development has been found in animal models^5^. Data in humans suggest impaired respiratory function in children and adolescents with low birthweight even though this was mainly related to prematurity^6,7^. Recent evidence has also found an association between being born small and a lower respiratory function in adult life^8^. Even though some data have been reported concerning changes in the pulmonary artery in FGR fetuses^9,10^, little is known about the vascular profile of the smaller vessels of the lung. Therefore, there is a need of prospective studies aiming to comprehensively assess fetal lung structure and function and bring a better understanding of the potential detrimental effect of growth restriction on fetal pulmonary development.

2D ultrasound is a widely available tool that allows the measurement of the pulmonary area and information regarding various locations within the lung vasculature can be obtained by applying pulse Doppler in the main pulmonary artery and its branches. Since, for technical reasons, pulmonary function tests cannot be performed in the human fetus, hyperoxygenation has been proposed as a useful tool to study fetal vascular reactivity^11,12^.

On the other hand, the use of artificial intelligence methods such as unsupervised machine learning algorithms (Multiple Kernel Learning (MKL) and K-means clustering) enables categorization of subjects with similar clinical parameters, hence potentially helping in the interpretation of results^13–15^. Likewise, lumped computational models have been proposed to recreate and better understand haemodynamic changes in the fetal circulation^16,17^. These models are based on the idea that flow in a tube is analogous to the current in an electrical circuit and flow properties such as viscosity, inertia and compliance can be modeled with resistors, inductors and capacitors respectively. Thus, an equivalent electric circuit of the fetal circulation can be obtained in order to estimate parameters such as vascular resistance, which cannot be directly measured by ultrasound.

Considering all of the above, we designed a prospective study to assess the fetal lung vasculature and its response to maternal hyperoxygenation in FGR fetuses. To this end, we used fetal pulmonary Doppler, both while mothers were breathing room air and after maternal hyperoxygenation; then, MKL and 0-lumped computational model were applied to estimate pulmonary and systemic vascular resistance in these two conditions.

## Methods

### Study population and design

This is a prospective cohort study of singleton pregnancies conducted in BCNatal (Hospital Clínic and Hospital Sant Joan de Déu) in Barcelona, Spain, from September 2018 to July 2021, including 97 FGR fetuses and 111 control fetuses with normal growth, matched by gestational age at ultrasound. FGR was defined as birthweight below 10th percentile. Cases were considered placenta-related if birthweight <3rd percentile, uterine Doppler PI >95th percentile and/or cerebroplacental ratio <5th percentile^18^. Fetuses with congenital malformations and/or structural or genetic abnormalities were excluded. The study protocol was reviewed and approved by the Institutional Review Board/Independent Ethics Committee from Hospital Clínic de Barcelona (HCB/2018/0838). All research was performed in accordance with relevant guidelines and regulations and in accordance with the Declaration of Helsinki (last update: 64th WMA General Assembly, Fortaleza, Brasil, october 2013). Written informed consent was obtained from all participants before entering the study. The study protocol included fetal ultrasound in baseline conditions and after maternal hyperoxygenation, collection of cord blood at delivery and review of the neonatal outcomes.

### Fetal ultrasound in baseline conditions

**Figure 1:**
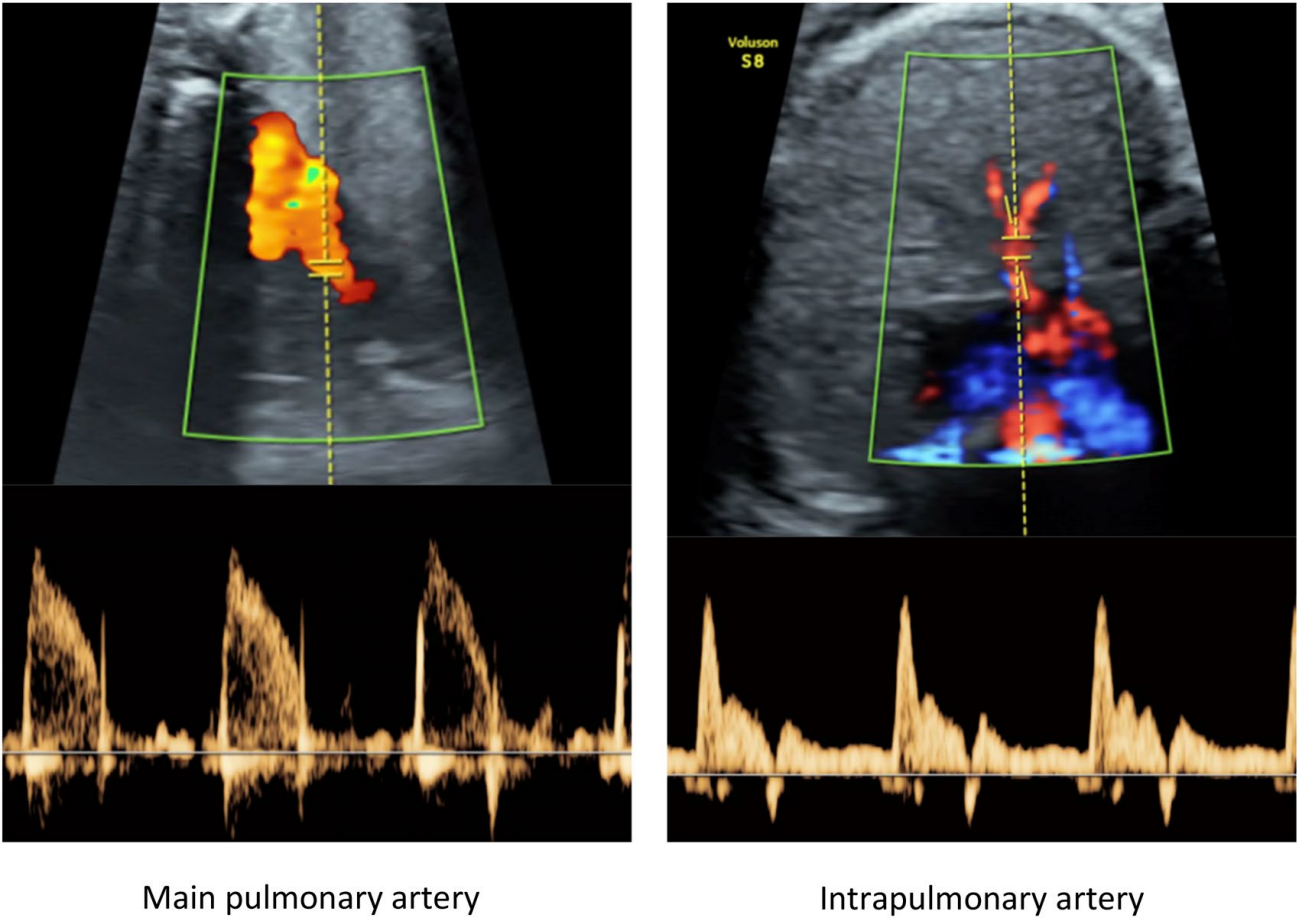
Illustrative images of fetal main pulmonary artery and intrapulmonary artery Doppler.

Fetal ultrasound was performed in all fetuses within a range from 24 to 37 weeks of gestation in baseline conditions (pregnant woman breathing room air). First, fetal weight was estimated using the formula suggested by Hadlock et al*.*^19^ and weight percentile was calculated using local standards^20^. Standard feto-placental Doppler included umbilical artery pulsatility index (PI), middle cerebral artery PI, cerebroplacental ratio, uterine arteries mean PI and ductus venosus PI following international guidelines^21,22^. In addition, fetal lung area was measured in a transverse plane of the fetal thorax at the level of a four-chamber view during ventricular diastole. The fetal spine was located at the bottom of the image to avoid acoustic shadow. Both right and left lung areas were measured by multiplication of the longest diameter of the lung by its longest perpendicular diameter^23^. Indexed values were obtained by dividing lung area by the estimated fetal weight. Images for fetal lung texture analysis were obtained in an axial four-chamber view of the thorax according to the methodology described by Palacio et al*.*^24^ then stored and analysed online using a bioinformatic tool, quantusFLM and its web interface (www.quantusflm.com; Transmural Biotech, Barcelona, Spain). Fetal pulmonary vasculature was also assessed using Doppler and 2D ultrasound. From a cross-sectional four-chamber view of the fetal heart and by slightly rotating the transducer towards the short-axis view of the heart, the bifurcation of the pulmonary artery was identified. Here the diameter of the artery was measured in 2D and Doppler was applied to get an image of the waveform allowing measurement of the PI, velocity time integral (VTI), peak systolic velocity and acceleration and ejection times of the main pulmonary artery^25^. Next, using the pulmonary veins as a landmark for the identification of the intrapulmonary artery on the side offering the best angle of insonation, pulsed Doppler was applied to obtain the PI, VTI and peak early-diastolic reverse flow (PEDRF) of the intrapulmonary artery^26,27^ (Fig. 1). Angle correction in the Doppler waveform acquisition was used when needed and kept <10° in all cases^22^.

### Maternal hyperoxygenation

In order to assess the fetal lung vascular reactivity to maternal hyperoxygenation, mothers breathed 100% oxygen using a face mask at a flow of 15 L/min during 10 min^28^. Fetal ultrasound of the main pulmonary and intrapulmonary arteries was again obtained after 10 min of maternal hyperoxygenation while maintaining the inspiratory flow of oxygen.

### Perinatal outcomes

Upon discharge of mother and newborn after delivery and hospitalization, medical records for both individuals were accessed to collect data regarding perinatal outcomes. Obstetric variables included gestational age at delivery, birthweight and preeclampsia occurrence. Considering the array of morbidity that can affect newborns, the different diagnoses were regrouped under the label of neonatal morbidity. This included hyperbilirrubinaemia, anaemia, neonatal thrombopenia, glucose metabolism disorders, sepsis, intraventricular haemorrhage, retinopathy, metabolic acidosis and persistent ductus arteriosus. Provided that respiratory morbidity is a special focus for this study it was also assessed separately. This variable included bronchopulmonary dysplasia, respiratory distress, pulmonary haemorrhage, transient tachypnoea of the newborn and bronchiolitis.

### Machine learning analysis of changes in fetal Doppler waveforms

The machine learning-based analysis of Doppler waveforms was performed using Unsupervised Multiple Kernel Learning (MKL)^29,30^ and logistic regression. Briefly, MKL allows simplified representations of a population to be obtained based on information from heterogeneous data sources (e.g. image, signal, structured data). Similarity (or kernel) matrices are built for each data source and combined, allowing a complex description of the similarity relations among all subjects to be obtained, weighted by different clinical descriptors. Finally, subjects are mapped to a simplified space where they are positioned close to each other if they present similarly in terms of all available data sources. In this paper, MKL was used to find a simplified representation of the population based on the main pulmonary and intrapulmonary arteries waveforms, both in baseline conditions and after maternal hyperoxygenation. Logistic regression was used to model the probability of the two classes (normally-grown and FGR) within the simplified space in baseline conditions. Subjects were then divided into two clusters according to the decision boundary of the logistic regression model. Changes in the main pulmonary and intrapulmonary arteries waveforms of a subject translate into displacements in the MKL space (Supplemental Fig. S1). Displacement vectors were computed for each subject to quantify changes after maternal hyperoxygenation. The component of the vectors normal to the decision boundary and pointing towards the controls’ decision region was isolated and used as an estimate of displacement towards normality (DTN). Finally, the magnitudes of DTN for the two clusters were compared.

### Computational model of the fetal pulmonary circulation

**Figure 2:**
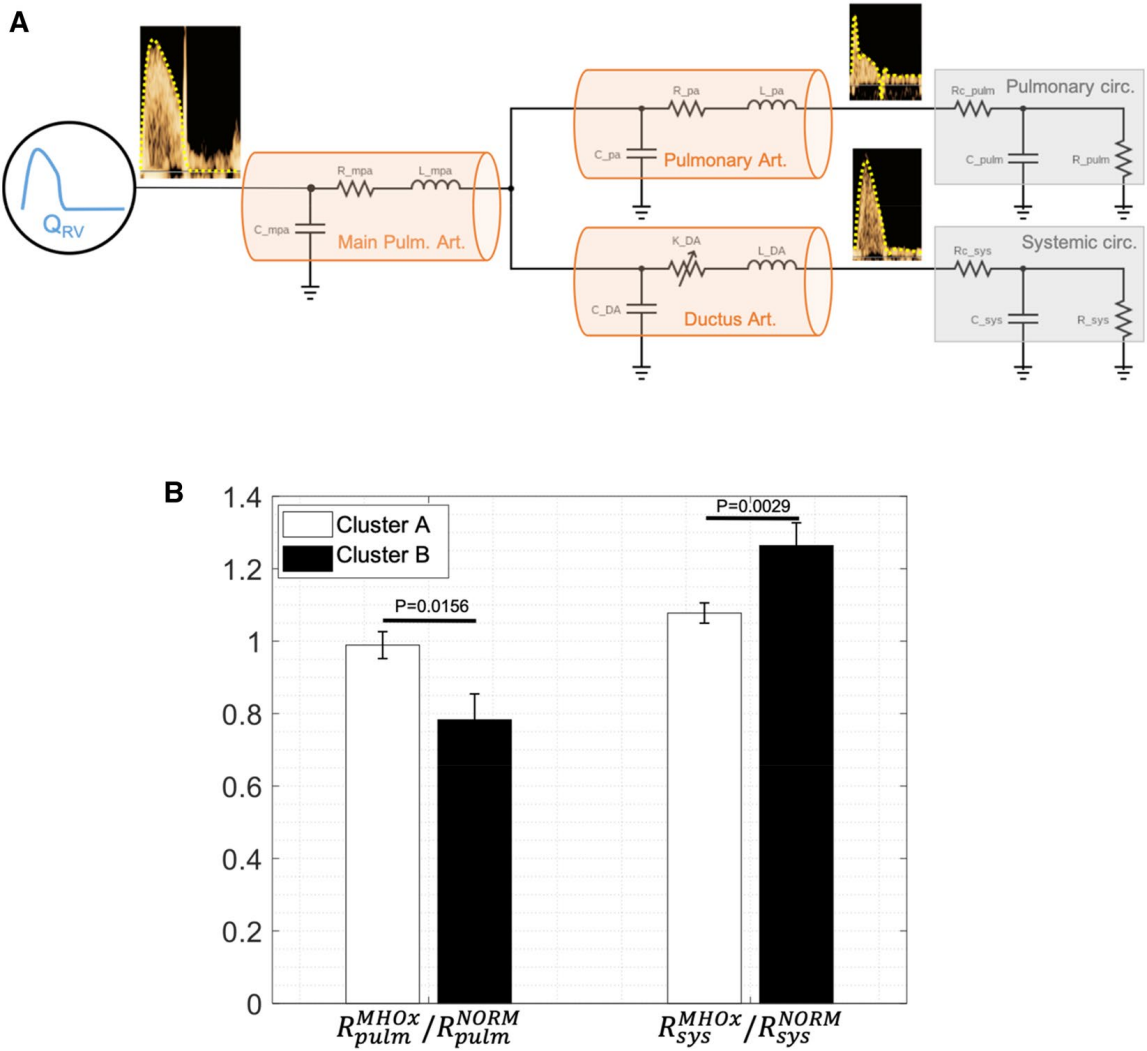
Schematic representation of the simplified computational model of the fetal pulmonary circulation (**A**) and results of the model-estimated pulmonary and systemic resistances in baseline conditions and after maternal hyperoxygenation (**B**). (**A**) Anatomical simplified configuration and equivalent lumped model of the fetal pulmonary circulation including one input, 3 arterial segments and 2 vascular beds. Q_RV_ represents main pulmonary artery input flow. Anatomical configuration composed of 3 arterial segments: (1) main pulmonary artery (2) intrapulmonary artery, and (3) ductus arteriosus. The electric circuit of the lumped model corresponding to the 3 arterial segments are highlighted in solid lines and include 1 resistor (R), 1 capacitor (C) and 1 inductor (L). The 2 blocks of vascular beds (pulmonary and systemic circulation) are highlighted in grey and they consist of a resistor Rc in series with a capacitor Cp in parallel with a resistor Rp. (**B**) Results of the ratio of the model-based pulmonary (Rlungs) and systemic (Rsys) resistance at maternal hyperoxygenation (MHOx) to model-based pulmonary and systemic resistances at maternal basal conditions (NORM) according to cluster A (white) and cluster B (black).

A simplified computational model of the pulmonary circulation was constructed (Fig. 2A) based on our previous fetal circulation model^16,31^. The simplified model of the pulmonary fetal circulation was implemented in Simulink, MATLAB (R2021a, The MathWorks Inc., Natick, MA) and consisted of one blood flow input, three arterial segments, and two vascular beds (Fig. 2A). Arterial segments (except the ductus arteriosus) were modelled as a capacitor in parallel with a resistor and an inductor. In the case of the ductus arteriosus, the resistor was replaced by a nonlinear resistor as described by Pennati et al.^32^ Peripheral vascular beds consisted of a three-element Windkessel model, including a resistor in series with a capacitor and a resistor in parallel, modelling the organ/tissue compliance and resistance respectively. The model was personalised using three Doppler velocity waveforms: main pulmonary artery, ductus arteriosus and intrapulmonary artery as well as pulmonary valve diameter, estimated fetal weight and gestational age of every subject. The estimated fetal weight and gestational age were used to calculate the nominal values of all electrical components as described in Garcia-Canadilla et al.^29^. Then, the measured main pulmonary artery velocity (*V*), both in baseline and hyperoxygenation conditions, together with the pulmonary valve diameter (*D*) were used to define the blood flow input of the model (*Q*), using the following equation: $Q=V\cdot \pi \cdot {(D/2)}^{2}$. The output of the model was the model-based blood velocities ($\widetilde{V}$) in the ductus arteriosus and intrapulmonary artery. Then, a constrained nonlinear optimisation algorithm^31^ minimising the normalised root mean square error (NRMSE) between model-based and measured blood velocity waveforms of the ductus arteriosus and intrapulmonary artery were used to estimate pulmonary and systemic resistances (R_pulm_ and R_sys_ in Fig. 2A, respectively) and compliances (C_pulm_ and C_sys_) both in baseline and in hyperoxygenation conditions. Therefore, the objective function to minimise, *J*, was defined as the sum of individual NRMSE as follows:

$$J= {\sum }_{i=DA, IPA}\frac{\sqrt{\frac{1}{N}\sum_{t=1}^{N}{\left({\widetilde{V}}_{i}\left(t\right)-{V}_{i}(t)\right)}^{2}}}{{\text{max}}\left({V}_{i}\left(t\right)\right)-{\text{min}}({V}_{i}\left(t\right))}$$

where *i* indicates one the two places of the fetal circulation where blood velocity was measured: ductus arteriosus and intrapulmonary artery; *N* is the number of time points of the velocity profile. More details on the personalisation process can be found in Garcia-Canadilla et al.^29^.

### Cord blood biomarkers

Umbilical cord blood samples were collected upon delivery and then centrifuged at 1500*g* for 10 min at 4 °C in order to separate serum from the cellular fraction. Serum samples were immediately stored at −80 °C until analysed. Serum concentrations of several specific pneumo-proteins such as surfactant protein A and D (SP-A and SP-D) and club cell protein 16 (CC16) were determined using either ELISA or Luminex^®^, following manufacturer’s instructions.

### Sample size estimation

Sample size calculation for this study was based on intrapulmonary artery PI, for which reference values have been already published in a normal population of fetuses^27^. Assuming an unknown but equal variance, an alpha error of 0.05, a beta error of 0.2 and a 1:1 allocation index, a sample of 140 individuals (70 per arm) was estimated to detect a 15% difference in the intrapulmonary artery PI.

### Statistical analysis

The statistical analysis was made using Student’s t-test or non-parametric Wilcoxon rank-sum test for continuous variables whereas chi-square or non-parametric Fisher’s exact tests were used for categorical variables. p-values presented in the tables refer to unpaired comparisons between the control and FGR groups, or between cluster A and B. Comparisons of the results obtained with ultrasound were adjusted by gestational age, and estimated fetal weight when appropriate, using multivariate linear regressions models. A p-value of <0.05 was considered statistically significant. Analyses were performed using Stata/IC 15.1 (StataCorp, College Station, TX) software.

## Results

### Characteristics of the study population

Baseline and perinatal characteristics of the study population are shown in Table 1. Maternal baseline characteristics were similar in the two study groups. As expected, FGR fetuses had lower birthweight and gestational age at delivery together with worse feto-placental Doppler, higher rates of preeclampsia, need for neonatal reanimation, admission to Neonatal Intensive Care Unit (NICU) and neonatal morbidity. There was only one case of perinatal mortality occurring in the FGR group.

**Table 1:** Maternal and perinatal characteristics of the study population.

	Controls	FGR	p-value
N	111	97	
Maternal characteristics			
Age (years)	33.8 ± 4.5	34.1 ± 5.7	0.668
Nulliparous (%)	40 (36.0)	34 (35.0)	0.944
Body mass index (kg/m^2^)	23.0 ± 4.8	23.2 ± 4.6	0.736
Use of assisted reproductive technologies (%)	14 (12.6)	14 (14.4)	0.747
Smokers (%)	15 (13.5)	9 (9.3)	0.404
Feto-placental ultrasound			
Gestational age at ultrasound (weeks)	33.1 ± 3.5	33.2 ± 3.5	0.886
Estimated fetal weight (g)	**2173 ± 734**	**1640 ± 667**	** <0.001**
Umbilical artery PI	**0.95 ± 0.19**	**1.18 ± 0.47**	** <0.001**
Middle cerebral artery PI	**1.95 ± 0.39**	**1.79 ± 0.40**	**0.013**
Cerebroplacental ratio	**2.07 ± 0.52**	**1.67 ± 0.54**	** <0.001**
Uterine arteries mean PI	**0.72 ± 0.19**	**1.07 ± 0.54**	** <0.001**
Ductus venosus PI	0.45 ± 0.17	0.52 ± 0.27	0.168
Perinatal data			
Gestational age at delivery (weeks)	**39.9 ± 1.1**	**37.3 ± 3.4**	** <0.001**
Prematurity (%)	**3 (2.7)**	**22 (22.7)**	** <0.001**
Birthweight (g)	**3338 ± 383**	**2191 ± 627**	** <0.001**
Birthweight percentile	**49 ± 29**	**4 ± 4**	** <0.001**
Female sex (%)	48 (43.2)	46 (47.4)	0.546
Preeclampsia (%)	**2 (1.8)**	**18 (18.6)**	** <0.001**
Need for neonatal reanimation (%)	**4 (3.6)**	**19 (19.6)**	** <0.001**
Admission to Neonatal Intensive Care Unit (%)	**2 (1.8)**	**7 (7.2)**	**0.035**
Neonatal morbidity (%)	**11 (9.9)**	**39 (40.2)**	** <0.001**

Data shown as mean ± SD or n (percentage).

Statistically significant different variables (< 0.05) variables are highlighted using bold text.

### Lung size, texture and biomarkers

FGR fetuses had a significantly smaller total lung area when compared with controls (Table 2). However, these differences disappeared when lung area was indexed by estimated fetal weight. Evaluation of the risk of respiratory morbidity by lung texture analysis failed to find differences between the two groups. Also, cord blood concentrations of CC16, SP-A and SP-D were similar in both control and FGR fetuses.

**Table 2:** Fetal lung size and texture by ultrasound and pulmonary cord blood biomarkers in the study populations.

	Controls	FGR	p-value
Fetal lung biometries and texture by ultrasound*			
* N*	*111*	*97*	
Gestational age at ultrasound (weeks)	33.1 ± 3.5	33.2 ± 3.5	0.886
Fetal total lung area (cm^2^)	**30.1 ± 17.2**	**25.3 ± 6.4**	**0.002**
Indexed fetal total lung area (cm^2^/kg)	14.9 ± 8.8	15.2 ± 4.00	0.757
Risk of respiratory morbidity by lung texture analysis (%)^¤^	27.2 ± 19.0	25.7 ± 18.6	0.582
Cord blood biomarkers^φ^			
* N*	*65*	*55*	
Club cell protein 16 (ng/mL)	12.1 ± 5.6	15.3 ± 6.2	0.053
Surfactant protein A (μg/mL)	57.6 ± 27.8	47.9 ± 16.2	0.184
Surfactant protein D (ng/mL)	39.4 ± 24.8	63.2 ± 33.2	0.077

Data shown as mean ± SD.

*p-value adjusted by gestational age at ultrasound.

^φ^p-value is adjusted by gestational age at delivery.

^¤^Individuals having received antenatal steroids were excluded (107 controls and 84 FGR fetuses were included).

Statistically significant differences (p <0.05) are highlighted in bold text.

### Fetal lung vasculature in baseline conditions

In baseline conditions (i.e., mothers breathing room air) and compared with controls, FGR fetuses showed significantly lower PI, VTI and peak systolic velocity, prolonged acceleration time and increased acceleration/ejection time ratio in the main pulmonary artery (Table 3). Likewise, their intrapulmonary artery PI was higher and their VTI lower. By contrast, the main pulmonary artery ejection time and diameter, and intrapulmonary artery PEDRF were similar among groups.

**Table 3:** Fetal pulmonary vascular results by ultrasound in baseline conditions and after maternal hyperoxygenation.

	Controls	FGR	p-value*
N	111	97	
Gestational age at ultrasound (weeks)	33.0 ± 3.5	33.2 ± 3.5	0.808
Pulmonary Doppler in baseline conditions			
Main pulmonary artery			
PI	**2.24 ± 0.22**	**2.16 ± 0.27**	**0.036**
Velocity time integral (cm)	**10.7 ± 3.5**	**9.15 ± 1.79**	** <0.001**
Peak systolic velocity (cm/s)	**78.5 ± 22.8**	**68.6 ± 12.4**	** <0.001**
Acceleration time (ms)	**41.5 ± 8.2**	**44.8 ± 12.6**	**0.026**
Ejection time (ms)	191.7 ± 10.2	188.7 ± 11.8	0.053
Acceleration/ejection time ratio	**0.22 ± 0.04**	**0.24 ± 0.06**	**0.006**
Diameter (mm)	5.66 ± 1.16	5.59 ± 1.26	0.978^†^
Intrapulmonary artery			
PI	**3.69 ± 1.12**	**4.22 ± 1.64**	**0.006**
Velocity time integral	**7.03 ± 2.02**	**6.22 ± 2.49**	**0.011**
Peak early-diastolic reverse flow (cm/s)	− 13.9 ± 4.8	− 13.9 ± 5.1	0.988
Pulmonary Doppler after maternal hyperoxygenation			
Main pulmonary artery			
PI	**2.25 ± 0.21**	**2.16 ± 0.25**	**0.004**
Velocity time integral (cm)	**10.5 ± 1.6**	**9.80 ± 1.70**	**0.003**
Peak systolic velocity (cm/s)	**77.2 ± 11.8**	**71.5 ± 11.9**	**0.001**
Acceleration time (ms)	**43.2 ± 7.5**	**46.2 ± 9.5**	**0.010**
Ejection time (ms)	194.6 ± 11.8	194.3 ± 10.5	0.868
Acceleration/ejection time ratio	**0.22 ± 0.04**	**0.24 ± 0.05**	**0.007**
Diameter (mm)	5.94 ± 1.13	5.68 ± 1.30	0.120^†^
Intrapulmonary artery			
PI	3.47 ± 1.14	3.40 ± 1.11	0.642
Velocity time integral (cm)	8.75 ± 3.11	7.30 ± 2.51	0.287
Peak early-diastolic reverse flow (cm/s)	− 12.5 ± 4.67	− 12.0 ± 3.75	0.425

Data shown as mean ± SD or n (percentage).

*PI* pulsatility index.

*p-value adjusted by gestational age at ultrasound.

^†^p-value adjusted by gestational age at ultrasound and estimated fetal weight.

Statistically significant different (p <0.05) variables are highlighted using bold text.

### Pulmonary vascular response to maternal hyperoxygenation

Table 3 shows the results of the pulmonary Doppler after 10 min of maternal hyperoxygenation. FGR persisted with lower main pulmonary artery PI, VTI and peak systolic velocity as well as prolonged acceleration time and increased acceleration/ejection time ratio as compared to controls, although most differences were less pronounced after maternal hyperoxygenation. Of note, intrapulmonary artery Doppler parameters were similar among groups. Delta changes of the main pulmonary artery VTI and intrapulmonary artery PI before and after hyperoxygenation were significantly greater in FGR when compared with controls (Table S1).

### Machine learning identified two clusters with dissimilar lung vascular reactivity

**Figure 3:**
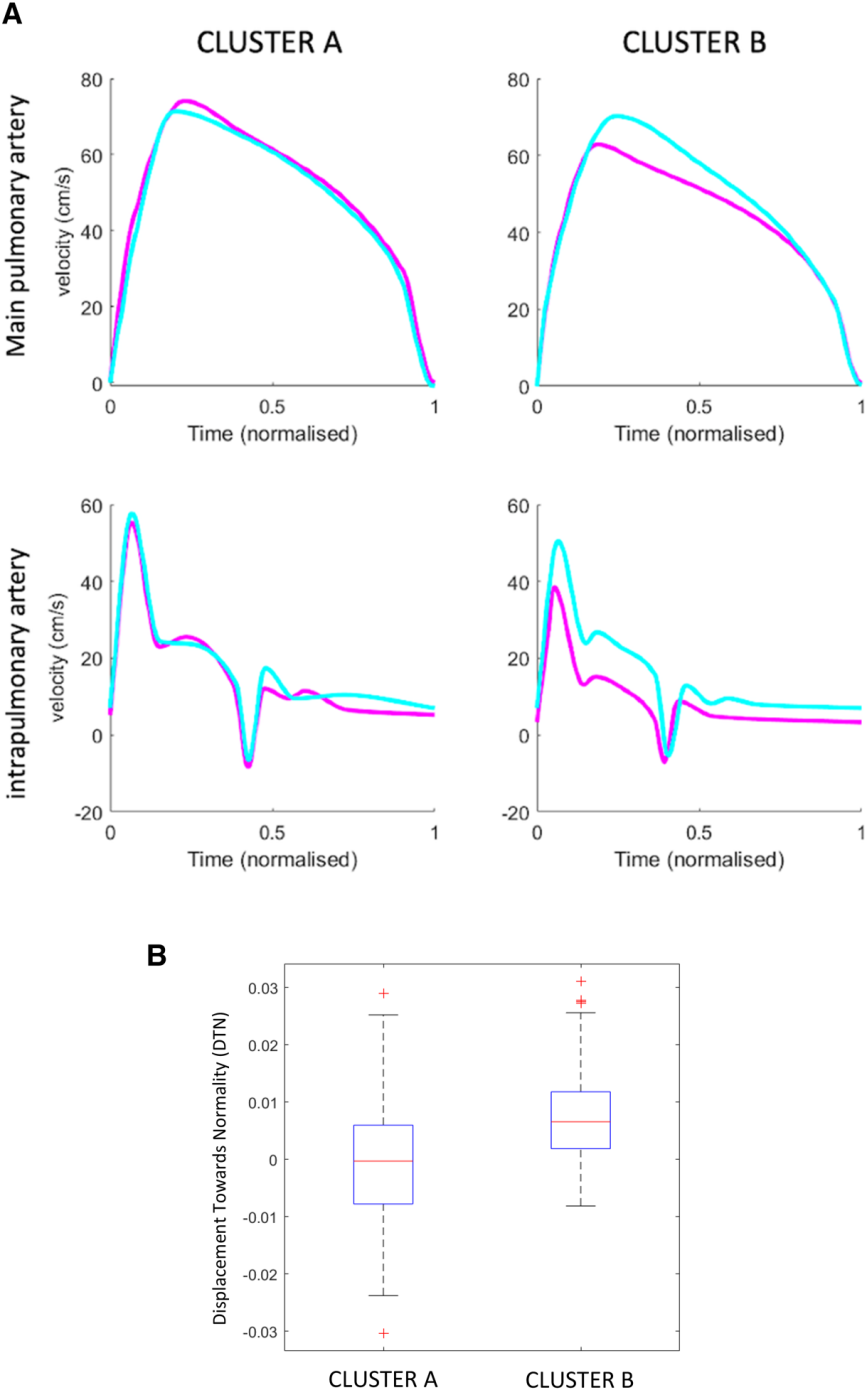
Machine learning analysis of changes in fetal lung Doppler waveforms in the study population identifying two different clusters. (**A**) Main and intrapulmonary Doppler traces from a representative individual in each cluster in baseline conditions and after maternal hyperoxygenation. Representative main pulmonary (top) and intrapulmonary (bottom) curves of each cluster in baseline conditions (violet) and after maternal hyperoxygenation (turquoise blue). Doppler traces from fetuses included in cluster A hardly change after maternal hyperoxygenation, while pulmonary Doppler traces significantly change after maternal hyperoxygenation in cluster B. Of note, after maternal hyperoxygenation fetuses in cluster B show a significant increase in peak velocity and delayed time-to-peak in the main pulmonary artery (top right panel) with also a significant increase in peak velocities of the intrapulmonary artery (bottom right). (**B**) Median and interquartile range values of displacement towards normality (DTN) distribution in the two identified clusters using MKL. Red lines indicate median values, boxes indicate interquartile range and whiskers extend from first and third quartiles to the most extreme values. Median DTN in cluster A is near 0, while DTN in cluster B is significantly higher (p-value <0.0001).

The logistic model’s decision boundary divided the population in two clusters according to their vascular reactivity to maternal hyperoxygenation (Figs. 3 and S1). Figure 3A shows the main pulmonary and intrapulmonary arteries traces representatives of an individual in cluster A and B in baseline conditions and after maternal hyperoxygenation. It illustrates that cluster A (containing mainly controls: 66% controls and 34% FGR) pulmonary waveforms showed minimal changes after maternal hyperoxygenation. Cluster B (containing mainly FGR cases: 67% FGR and 33% controls) showed pronounced changes after maternal hyperoxygenation, approximating the counterparts of cluster A, with increased peak velocities in both pulmonary arteries, and also prolonged acceleration time in the main pulmonary artery after maternal hyperoxygenation. Displacement towards normality was significantly higher in cluster B when compared to cluster A (Fig. 3B).

Cluster B included mainly FGR cases and associated worse perinatal outcomes, with lower gestational age at delivery, birthweight and birthweight percentile, and higher rates of preeclampsia, need for neonatal reanimation and neonatal morbidity (Table 4). There was also a non-significant trend to higher rates of respiratory morbidity. To note, cluster A contained mainly controls and mild FGR, with the exception of 5 severe FGR cases. Table S2 depicts the characteristics of these 5 cases, that showed no capacity to react after maternal hyperoxygenation, all of them with abnormal feto-placental Doppler requiring delivery before 32 weeks of gestation, need of neonatal reanimation and associated neonatal respiratory morbidity. It also included the only one case of neonatal death of this study.

**Table 4:** Perinatal characteristics of the study population subdivided according to the two clusters identified using MKL applied on feto-placental Doppler at baseline and after maternal hyperoxigenation.

	Cluster A (n = 150)	Cluster B (n = 36)	p-value
Gestational age at delivery (weeks)	**39.1 ± 2.2**	**37.3 ± 3.6**	**0.006**
Prematurity (%)	**10 (6.67)**	**11 (30.6)**	** <0.001**
Birthweight (g)	**2968 ± 650**	**2270 ± 886**	** <0.001**
Birthweight percentile	**33 ± 31**	**13 ± 24**	** <0.001**
Female sex (%)	72 (48.0)	15 (41.7)	0.494
Preeclampsia (%)	**6 (4.0)**	**10 (27.8)**	** <0.001**
Need for neonatal reanimation (%)	**12 (8.0)**	**8 (22.2)**	**0.029**
Admission to Neonatal Intensive Care Unit (%)	6 (4.0)	3 (8.33)	0.183
Neonatal respiratory morbidity (%)	10 (6.7)	3 (8.3)	0.722
Neonatal morbidity (%)	**25 (16.7)**	**15 (41.7)**	**0.001**

Data shown as mean ± SD or n (percentage).

Statistically significant different variables (<0.05) variables are highlighted using bold text.

### The 0-lumped computational model estimated a different fetal lung resistance response in the two clusters

The simplified computational model of the fetal pulmonary circulation was personalised using Doppler data from 91 fetuses. Pulmonary circulation resistances before and after maternal hyperoxygenation were estimated, where 1 indicates no change, >1 indicates an increase and <1 indicates a decrease. The ratios of pulmonary and systemic circulation resistances were significantly different between cluster A and cluster B (ratio Rlung_O2_/Rlungs_norm_ cluster A 0.98 ± 0.33 vs cluster B 0.78 ± 0.28, p = 0.0156; ratio Rtotal_O2_/Rtotal_norm_ cluster B 1.26 ± 0.24 vs cluster A 1.07 ± 0.21, p = 0.0029) (Fig. 2B), with cluster A having values closer to 1. No relationship with gestational age was observed (data not shown). Absolute values of pulmonary and systemic vascular resistances and compliances are shown in Supplementary Table S3.

## Discussion

### Summary of main findings

Our results show that: *(1)* growth restricted fetuses have evidence of abnormal pulmonary vasculature at baseline and respond differently to maternal hyperoxygenation than normal growth individuals; and, *(2)* machine learning analysis and the computational model identify a subgroup of FGR fetuses with more pulmonary vascular reactivity to maternal hyperoxygenation which, importantly, had better perinatal outcomes.

### Pulmonary haemodynamics at baseline, breathing room air

Our Doppler data suggest a different pulmonary vasculature in the FGR group. Indeed, the main pulmonary artery had a lower PI and peak systolic velocity among FGR individuals and its velocity time integral was also reduced. However, these changes in lung vasculature do not seem to be related to lung size or parenchymal structure as indexed fetal lung area, ultrasound texture and blood biomarkers were similar between groups. Inconsistent results are found in the literature when it comes to pulmonary Doppler parameters. Some authors described an increased PI in the main pulmonary artery in FGR fetuses although the sample size was particularly small (only 7 subjects)^33^ whereas others identified lower PI values both in the main and right and left pulmonary arteries in FGR individuals^34^, with our findings being in line with the latter. Also, lower values of the acceleration/ejection time ratio have been negatively associated with fetal lung immaturity and risk of respiratory distress syndrome in fetuses with normal growth^25,35^. Our data is also consistent with previous studies reporting increased intrapulmonary artery PI in FGR and related to vascular hypoxia^36^. Previous data from congenital diaphragmatic hernia (CDH) suggest increased intrapulmonary PI and PEDRF associated to poorer prognosis^37,38^. Our results go partially in the same direction but no differences between groups were found in the PEDRF results, thus suggesting that the physiopathology of the injury in FGR differs from the one occurring in CDH. Also, an increase in the pulmonary veins PI has been described in FGR fetuses, suggesting abnormalities of the lung vascular bed even though these vessels can also be strongly influenced by pathology of the left atrium and/or an increase in pressure at this level^39^. In a nutshell, the differences that have been described could either reflect primary changes in the lung vasculature or be secondary to alterations in fetal cardiac function.

### Response to maternal hyperoxygenation

In our study, delta changes of the pulmonary Doppler before and after maternal hyperoxygenation were significantly greater in FGR when compared with controls. To date, all previous studies evaluated the pulmonary vasculature in terms of PI and some have tested its reactivity to oxygen administration. Some authors have found an association between maternal oxygen administration and a decrease in the pulmonary vessels PI^34^. Longitudinal data have shown that pulmonary vascular reactivity to maternal oxygen administration is acquired with advancing gestational age and that little changes are observed before mid-third trimester^11,40^. However, it has also been described that oxygen response is variable among fetuses with normal development and that its clinical application may be debatable^40^. In our study, the only vessel to show significant differences between study groups in terms of delta change in its PI after maternal hyperoxygenation was the intrapulmonary artery. The lack of differences in the main pulmonary artery PI could be explained by the wide range of gestational age at which the ultrasounds were performed (many occurring before mid-third trimester) and the fact that all were single observations with no longitudinal follow-up. In observations carried out in fetuses suffering from conditions leading to lung hypoplasia, such as CDH, an association between pulmonary vascular reaction to maternal hyperoxygenation and the outcome was found. In this case, fetuses more reactive to oxygen administration were more prone to survival^12,41^. In order to try to understand the underlying mechanisms of these changes, we decided to apply machine learning tools and to use a computational model. Machine learning enabled identification of two different pulmonary responses to maternal hyperoxigenation: cluster A containing mainly controls who showed no/minor changes in pulmonary Doppler traces after maternal hyperoxygenation; and cluster B containing mainly FGR cases whose main and intrapulmonary Doppler traces significantly changed with a greater approximation to normality when oxygen was administered to the mother. More precisely, mean velocities increased in both vessels after oxygen administration and a delay was observed in time to peak velocity, thus extending the length of the acceleration time, probably due to wave reflections. This variation could be explained by an preexisting status of hypoxic pulmonary vasoconstriction^42^ in FGR individuals. Interestingly, it appears that two different types of response among FGR individuals might exist: on the one hand, the majority of cases who have different baseline pulmonary Doppler parameters and who show a strong response to oxygen administration, hence “normalising” their waveforms by bringing them closer to subjects with normal growth. On the other hand, we find the most severe cases (5 fetuses) who may have lost their ability to respond or their compensation mechanisms and who have a worse prognosis.

The addition of the computational model enabled the estimation of the pulmonary vascular resistance which otherwise cannot be measured directly. The model estimated a greater change of the lung vascular resistance in the group where FGR was predominant, that is, a greater reactivity to the administration of oxygen.

### Potential relation to neonatal morbidity and long-term consequences in lung function later in life

FGR individuals have more neonatal morbidity and need for neonatal reanimation with a tendency towards more respiratory distress. This is mainly observed in the FGR cases from the more reactive cluster B but also in the more severe cases of cluster A who show no reactivity. Our study included both preterm and term born fetuses. The respiratory outcomes among preterm-born individuals agree with earlier findings in subjects born SGA and preterm^43^. On the other hand, Lio et al. observed significant differences in terms of development of bronchopulmonary dysplasia between FGR and non-FGR very preterm-born babies (≤30 weeks)^44^. Given the disparity in terms of early respiratory outcomes, the actual relevance of being born very or extremely-preterm vs moderate or late-preterm on the development of pulmonary morbidity in FGR fetuses still needs to be elucidated.

Low birthweight has previously been associated with altered respiratory function both in children^6^ and adult cohorts^45^. However, most perinatal data was collected retrospectively in these individuals and prematurity was also quite prevalent. Interestingly, some new data from a longitudinal cohort have shown that adult individuals born with low birthweight had a reduced lung function which could potentially be linked to lungs being smaller^8^. All things considered, the effect of birthweight alone may not have been explored to its full extent. A meta-analysis from den Dekker et al. showed impaired features in lung function in children who were born SGA which differed from the altered parameters in the ones born preterm^46^. Another study found evidence supporting that FGR is an independent factor for poorer lung function in children born very preterm^7^. On the other hand, lung function tests performed in large population-based cohorts^47,48^ suggest that up to 10% of early adults have a low respiratory function. Authors point towards factors occurring earlier in life, that would have the potential of being modified or prevented, as a plausible negative influence on lung development. These data are consistent with our recently published data suggesting reduced FEV_1_ and exercise capacity in adults born SGA at term^49,50^. Levels of lung-specific biomarkers have been analysed in a postnatal cohort of young individuals and an association between lower levels of CC16 and a lower FEV_1_ was observed^51^. We could not replicate these findings in our population but prenatal life could be too early to establish such a link in these individuals.

## Strengths and limitations

This study has some strengths and limitations that merit comment. The study is based on the construction of a prospective cohort of individuals using very strict selection criteria. A comprehensive characterisation of the participants was achieved with the use of various diagnostic tools including ultrasound but also a thorough post-processing and machine learning analysis. Computational models are being increasingly used by medical research due to their ability to help understanding the pathophysiological mechanisms of complex diseases. A model developed by our group has already been used to study the fetal circulation in FGR and placental resistance^16^. The application of an adapted model has allowed us to find consistent results with those found with the Doppler ultrasound assessment both in baseline conditions and after maternal hyperoxygenation. On the other hand, we acknowledge some limitations to this study. FGR contains a variety of individuals within the spectrum of the disease and the remarkable characteristics of a subgroup of individuals might have been diluted when analysed as a whole cohort. Prematurity is also a strong potential confounder in terms of neonatal respiratory outcomes and, even though we adjusted our results for this variable, studies designed specifically to study the independent contributions of preterm birth and FGR may be needed. The use of steroids could also constitute a source of bias but the study was not powered to detect their effect in our population. The intrapulmonary artery was measured either on the right or left according to the side providing a better insonation angle instead of systematically measuring the same artery for all participants. We acknowledge that this could add some variability as these arteries may have different Doppler characteristics. Also, fetuses were assessed only once during the participation in the study and the lack of longitudinal data could limit their interpretation.

## Conclusions and clinical relevance of findings

Our data suggest that there might be a different pulmonary vasculature in growth restricted fetuses. We also identify two distinct types of response to oxygen -minimal response or high vasoreactivity- that may be associated to different perinatal outcomes. Future studies are warranted to better study the relationship between fetal pulmonary vascular response to oxygen and respiratory outcomes, especially with means of repeated observations on the same individuals. Prenatal Doppler might have the ability to potentially identify those fetuses at higher risk of developing pulmonary disease in the future. The verification of such link could prompt the development of preventive and/or early therapeutic strategies in FGR for the improvement of their lung function.

### Supplementary Information

Supplementary Information.

## Data Availability

Data collected for the study are available upon reasonable request to the corresponding author.
