# Author Correction: Pulmonary vascular reactivity in growth restricted fetuses using computational modelling and machine learning analysis of fetal Doppler waveforms

**DOI:** 10.1038/s41598-024-58858-2

**Published:** 2024-04-09

**Authors:** Kilian Vellvé, Patricia Garcia‑Canadilla, Mariana Nogueira, Lina Youssef, Angela Arranz, Ayako Nakaki, David Boada, Isabel Blanco, Rosa Faner, Francesc Figueras, Àlvar Agustí, Eduard Gratacós, Francesca Crovetto, Bart Bijnens, Fàtima Crispi

**Affiliations:** 1 BCNatal Fetal Medicine Research Center (Hospital Clínic and Hospital Sant Joan de Déu), University of Barcelona, Sabino Arana 1, 08028 Barcelona, Spain; 2 Institut d’Investigacions Biomèdiques August Pi I Sunyer (IDIBAPS), Barcelona, Spain; 3 Interdisciplinary Cardiovascular Research Group, Institut de Recerca Sant Joan de Déu, Esplugues de Llobregat, Barcelona, Spain; 4 Pneumology Department, Respiratory Institute, Hospital Clínic, University of Barcelona, Barcelona, Spain; 5 Centre for Biomedical Research On Respiratory Diseases (CIBER-ES), Madrid, Spain; 6 Cathedra Salud Respiratoria, University of Barcelona, Barcelona, Spain; 7 Centre for Biomedical Research on Rare Diseases (CIBER-ER), Madrid, Spain; 8 ICREA, Barcelona, Spain

Correction to: *Scientific Reports* 10.1038/s41598-024-54603-x, published online 11 March 2024

The original version of this Article contained an error in the Funding section.

“This article was funded by European Commission (Framework Agreement number: 2013-0040), 'la Caixa' Foundation (LCF/PR/GN14/10270005), Instituto de Salud Carlos III (PI17/1515), Cerebra Foundation for the Brain Injured Children, Carmarthen, Wales, UK, Agència de Gestió d'Ajuts Universitaris i de Recerca (2017 SGR nº1531), Ministerio de Educación y Ciencia, Spain (FPU17/05579), CIBER Enfermedades Raras, Spain (ERPR04G719/2016).”

now reads:

“This article was funded by European Commission (Framework Agreement number: 2013-0040), 'la Caixa' Foundation (LCF/PR/GN14/10270005), Instituto de Salud Carlos III (INT21/00027, PI18/00073, PI20/00246), Cerebra Foundation for the Brain Injured Children, Carmarthen, Wales, UK, Agència de Gestió d'Ajuts Universitaris i de Recerca (2017 SGR nº1531), Ministerio de Educación y Ciencia, Spain (FPU17/05579), CIBER Enfermedades Raras, Spain (ERPR04G719/2016).”

The original Article has been corrected.

