# Supplementary material for: Pulmonary vascular reactivity in growth restricted fetuses using computational modelling and machine learning analysis of fetal Doppler waveforms
Source: Sci Rep. 2024 Mar 11;14:5919. doi: 10.1038/s41598-024-54603-x (PMC10928161; doi:10.1038/s41598-024-54603-x)
Supplement: Supplementary file 1 — Supplementary Information. [file 41598_2024_54603_MOESM1_ESM.docx]

**SUPPLEMENTARY MATERIAL**

**Pulmonary vascular reactivity in growth restricted fetuses using computational modelling and machine learning analysis of fetal Doppler waveforms**

**SUPPLEMENTARY TABLES**

**Table S1.** Delta change of pulmonary Doppler before and after maternal hyperoxygenation in the study populations.

|  | **Controls**  (n=111) | **FGR**  (n=97) | ***p-value*** |
| --- | --- | --- | --- |
| **Main pulmonary artery** | | | |
| Δ PI | 0.02 ± 0.26 | -0.00 ± 0.26 | *0.541* |
| **Δ velocity time integral** | **0.06 ± 1.26** | **0.66 ± 1.40** | ***0.001*** |
| Δ peak systolic velocity (cm/s) | 0.52 ± 9.77 | 2.95 ± 9.46 | *0.076* |
| Δ acceleration time (ms) | 1.93 ± 8.28 | 1.37 ± 10.89 | *0.672* |
| Δ ejection time (ms) | 3.22 ± 12.77 | 5.38 ± 12.55 | *0.225* |
| Δ diameter (mm) | 0.26 ± 0.87 | 0.10 ± 1.08 | *0.092* |
| **Intrapulmonary artery** | | | |
| **Δ PI** | **-0.22 ± 1.35** | **-0.85 ± 1.59** | ***0.002*** |
| Δ velocity time integral | 1.72 ± 13.50 | 1.12 ± 2.87 | *0.675* |
| Δ peak early-diastolic reverse flow (cm/s) | 1.45 ± 5.10 | 1.90 ± 5.05 | *0.525* |
| Δ: variation  Data shown as mean ± SD.  Mean difference between variables in baseline conditions and after maternal hyperoxygenation were calculated using a paired sample t-test. Means were compared using a multivariate linear regression adjusting by gestational age at ultrasound. For the main pulmonary artery diameter, means were compared using a multivariate linear regression adjusting by gestational age at ultrasound and estimated fetal weight.  Significantly different (p<0.05) variables are highlighted using bold text | | | |

**Table S2.** Characteristics of the five severe FGR cases included in cluster A

|  | Subject 1 | Subject 2 | Subject 3 | Subject 4 | Subject 5 |
| --- | --- | --- | --- | --- | --- |
| *Fetal ultrasound in basal conditions* |  |  |  |  |  |
| Umbilical artery PI | 1.95 | 1.66 | 2.54 | REDF | REDF |
| Middle cerebral artery PI | 1.29 | 1.50 | 1.34 | 1.43 | 1.09 |
| Cerebroplacental ratio | 0.66 | 0.90 | 0.53 | *NA* | *NA* |
| Ductus venosus PI | 0.32 | 1.18 | 0.58 | 1.18 | Reversed a-wave |
| Main pulmonary artery PI | 2.67 | 2.49 | 2.19 | 1.98 | 2.05 |
| Main pulmonary artery PSV (cm/s) | 101.6 | 77.7 | 78.8 | 71.8 | 55.0 |
| Main pulmonary artery AT/ET ratio | 0.25 | 0.22 | 0.17 | 0.20 | 0.23 |
| Intrapulmonary artery PI | 4.52 | 3.57 | 3.35 | 2.52 | 2.98 |
| *Fetal ultrasound after maternal hyperoxygenation* |  |  |  |  |  |
| Main pulmonary artery PI | 2.29 | 2.18 | 2.13 | 1.92 | 1.84 |
| Main pulmonary artery PSV (cm/s) | 97.2 | 81.1 | 87.9 | 60.2 | 46.1 |
| Main pulmonary artery AT/ET ratio | 0.25 | 0.21 | 0.18 | 0.20 | 0.31 |
| Intrapulmonary artery PI | 2.99 | 3.24 | 2.32 | 2.35 | 2.49 |
| *Perinatal results* |  |  |  |  |  |
| Gestational age at delivery (weeks) | 31.3 | 30.0 | 30.1 | 30.0 | 26.9 |
| Sex | Male | Male | Female | Male | Male |
| Birtweight (g) | 1340 | 980 | 1240 | 960 | 395 |
| Birtweight percentile | 2 | <1 | 5 | <1 | <1 |
| Need of reanimation | Yes | Yes | Yes | Yes | Yes |
| NICU admission | Yes | Yes | No | No | Yes |
| Neonatal morbidity | Yes | Yes | Yes | Yes | Yes |
| Neonatal respiratory morbidity | Yes | Yes | Yes | Yes | Yes |
| Perinatal death | No | No | No | No | Yes |

REDF: reverse end-diastolic flow; *NA*: not applicable

**Table S3**. Pulmonary and systemic resistance and compliance of the individuals in Cluster A and Cluster B.

|  | **Cluster A**  (n=76) | **Cluster B**  (n=15) | ***p-value*** |
| --- | --- | --- | --- |
| R_pulm_ NORMOXIA (mmHg·s·mL^-1^) | 69.16 ± 45.87 | 101.21 ± 89.50 | *0.1209* |
| R_pulm_ HYPEROXIA (mmHg·s·mL^-1^) | 65.01 ± 42.50 | 82.98 ± 97.22 | *0.8390* |
| R_sys_ NORMOXIA (mmHg·s·mL^-1^) | 9.81 ± 7.23 | 14.50 ± 15.64 | *0.0812* |
| **R_sys_ HYPEROXIA (mmHg·s·mL^-1^)** | **10.30 ± 7.30** | **17.99 ± 19.08** | ***0.0166*** |
| C_pulm_ NORMOXIA (mL·mmHg^-1^) | 0.008 ± 0.012 | 0.007 ± 0.006 | *0.8547* |
| C_pulm_ HYPEROXIA (mL·mmHg^-1^) | 0.006 ± 0.007 | 0.007 ± 0.006 | *0.5996* |
| C_sys_ NORMOXIA (mL·mmHg^-1^) | 0.078 ± 0.059 | 0.060 ± 0.048 | *0.2791* |
| **C_sys_ HYPEROXIA (mL·mmHg^-1^)** | **0.074 ± 0.059** | **0.035 ± 0.031** | ***0.0129*** |
| Data shown as mean ± SD  Statistically significant different variables (<0.05) variables are highlighted using bold text. | | | |

**SUPPLEMENTARY FIGURES**

**Figure S1.** Details of the machine learning analysis of changes in fetal lung Doppler waveforms in the study population identifying two different clusters.

**Figure S1A**. Multiple Kernel Learning (MKL) space of the control (green dots) and FGR (blue dots) individuals according to their main pulmonary and intrapulmonary arteries Doppler curves change after maternal hyperoxygenation displaying total displacement (arrow).


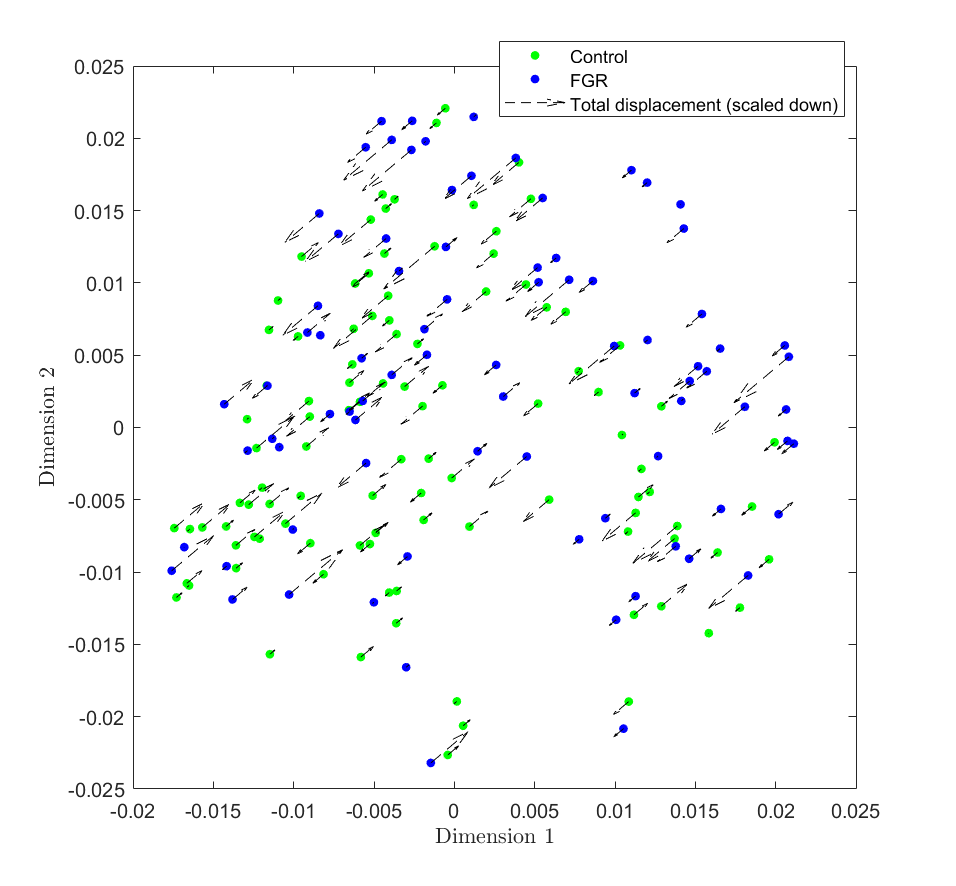


**Figure S1B**. Multiple Kernel Learning (MKL) space of the individuals according to their main pulmonary and intrapulmonary arteries Doppler curve change after maternal hyperoxygenation. Most individuals in cluster B (located to the right of the decision boundary line) were identified as FGR (blue dots), while cluster A (right) was mainly composed by controls (green dots).


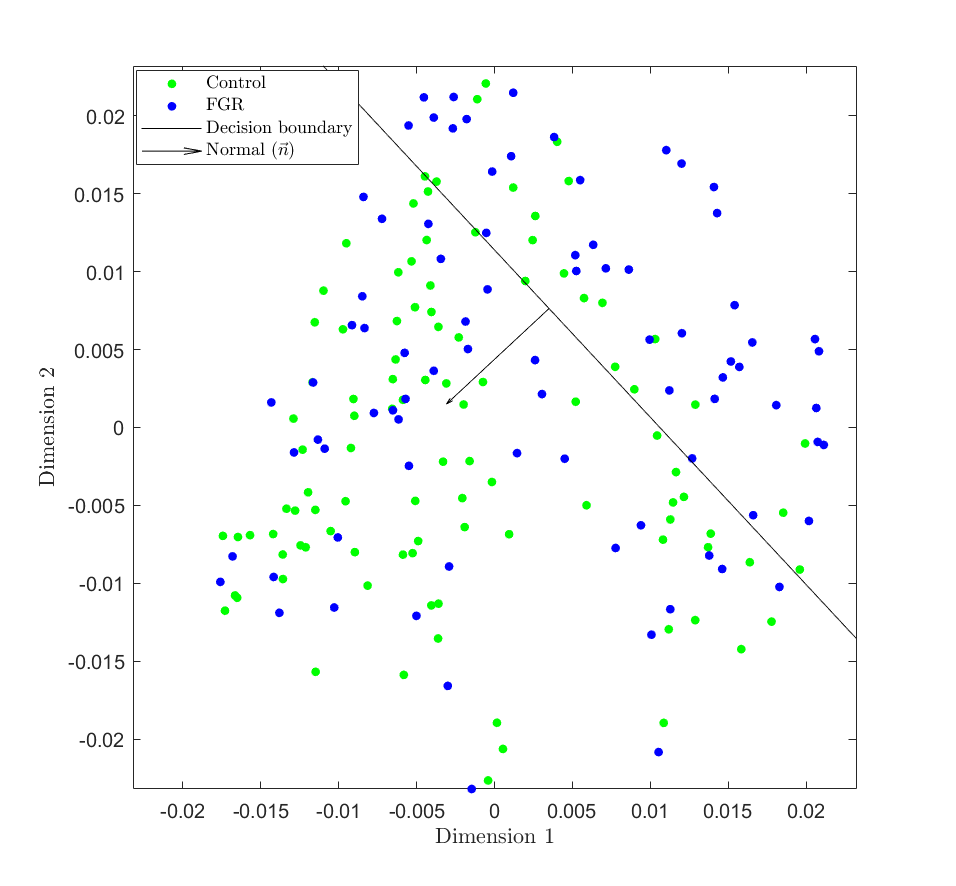


CLUSTER B

(hyperreactive)

CLUSTER A

(minimally reactive)
